# Prognostic Value of Inflammatory Status in Patients with Acute Coronary Syndromes: A Single-Center Experience

**DOI:** 10.3390/jcm15082852

**Published:** 2026-04-09

**Authors:** Ruxandra-Maria Băghină, Simina Crișan, Silvia Luca, Oana Pătru, Mihai-Andrei Lazăr, Cristina Văcărescu, Marian Morenci, Alina-Gabriela Negru, Constantin-Tudor Luca, Dan Gaiță

**Affiliations:** 1 Cardiology Department, “Victor Babes” University of Medicine and Pharmacy, Eftimie Murgu Square 2, 300041 Timisoara, Romania; ruxandra.croicu@umft.ro (R.-M.B.); silvia.luca@umft.ro (S.L.); oana.patru@umft.ro (O.P.); lazar.mihai@umft.ro (M.-A.L.); cristina.vacarescu@umft.ro (C.V.); alinanegru@umft.ro (A.-G.N.); constantin.luca@umft.ro (C.-T.L.); dan.gaita@umft.ro (D.G.); 2 Institute of Cardiovascular Diseases Timisoara, 13A Gheorghe Adam Street, 300310 Timisoara, Romania; 3 Research Center, Institute of Cardiovascular Diseases Timisoara, 13A Gheorghe Adam Street, 300310 Timisoara, Romania; 4 Doctoral School, “Victor Babes” University of Medicine and Pharmacy, 300041 Timisoara, Romania; 5 Doctoral School of Biomedical Sciences, Faculty of Medicine and Pharmacy, University of Oradea, 410087 Oradea, Romania; morenci.marian@student.uoradea.ro

**Keywords:** inflammation, myocardial infarction, C-reactive protein

## Abstract

**Background/Objectives**: Acute coronary syndromes (ACS) encompass a spectrum of clinical entities from unstable angina to non–ST-segment elevation myocardial infarction (NSTEMI) and ST-segment elevation myocardial infarction (STEMI), all associated with significant morbidity and mortality. Inflammation plays a central role in the pathophysiology of ACS, contributing to atherosclerotic plaque destabilization, myocardial injury, and adverse clinical outcomes. Inflammatory biomarkers, together with N-terminal pro–B-type natriuretic peptide (NT-proBNP), are increasingly used for risk stratification, yet their prognostic value across different ACS presentations remains unclear. This study aimed to assess the prognostic value of inflammatory status in patients with acute coronary syndromes in a single-center cohort. **Methods**: This prospective observational study included 100 consecutive patients with ACS and elevated inflammatory biomarkers, enrolled in 2024–2025 at a tertiary cardiovascular center. Inflammatory status was assessed by using C-reactive protein (CRP), neutrophil-to-lymphocyte ratio (NLR) and systemic immune-inflammation index (SII); NT-proBNP was also measured. The primary endpoint was in-hospital MACE, defined as cardiovascular death, recurrent myocardial infarction, stroke, urgent coronary revascularization, or acute heart failure requiring escalation of therapy. Multivariable logistic regression and ROC analyses were performed. **Results**: Among the 100 ACS patients, half experienced in-hospital MACE. Compared with those without events, patients with MACE were older (*p* = 0.003) and had higher inflammatory biomarkers—CRP (*p* < 0.001; strongest association), NLR (*p* = 0.030), and SII (*p* = 0.042)—as well as higher NT-proBNP (*p* = 0.002). Patients with MACE also showed reduced renal function (*p* < 0.001) and lower left ventricular systolic function, reflected by reduced LVEF (*p* = 0.001), indicating concomitant renal impairment and ventricular dysfunction. Hypertension was more prevalent in the MACE group (*p* = 0.028), and new-onset atrial fibrillation was significantly more common among these patients (*p* < 0.001). In multivariable analysis, LVEF emerged as an independent predictor of short-term outcomes (OR 0.934 per 1% increase; *p* = 0.047). **Conclusions**: Inflammatory activation appears closely linked to the occurrence of in-hospital adverse events in patients with acute coronary syndromes. While left ventricular ejection fraction remained an independent determinant of short-term outcomes, inflammatory biomarkers may provide complementary insight into the inflammatory burden accompanying ACS.

## 1. Introduction

Acute coronary syndromes (ACS) remain one of the leading causes of morbidity and mortality worldwide, despite significant advances in diagnostic and therapeutic strategies. ACS encompasses a spectrum of clinical entities, including ST-segment elevation myocardial infarction, non–ST-segment elevation myocardial infarction and unstable angina, which result from the rupture or erosion of atherosclerotic plaques followed by thrombus formation and myocardial ischemia. Early risk stratification in these patients is essential for guiding therapeutic decisions and improving clinical outcomes [1].

Inflammation plays a central role in the pathophysiology of atherosclerosis and its acute complications. The inflammatory cascade contributes not only to plaque formation and progression but also to plaque destabilization and thrombotic events leading to ACS. Consequently, increasing attention has been directed toward inflammatory biomarkers that may reflect disease activity and help identify patients at higher risk of adverse cardiovascular events. Several inflammatory markers derived from routine laboratory tests have emerged as potential prognostic indicators in ACS. C-reactive protein, a well-established marker of systemic inflammation, has been associated with plaque instability and worse cardiovascular outcomes. Similarly, the NLR has been proposed as an accessible indicator of systemic inflammatory response and has demonstrated prognostic value in patients with acute coronary syndromes [2]. More recently, the systemic immune-inflammation index, which integrates neutrophil, lymphocyte, and platelet counts, has gained attention as a comprehensive marker of inflammatory and immune status [3,4]. In addition to inflammatory biomarkers, markers of myocardial stress and cardiac dysfunction, such as N-terminal pro–B-type natriuretic peptide, have been widely recognized for their prognostic significance in patients with ACS. Elevated NT-proBNP levels have been associated with an increased risk of heart failure, recurrent ischemic events, and mortality [5].

Although these biomarkers have been individually investigated in various cardiovascular conditions, their combined prognostic role in patients presenting with different forms of acute coronary syndromes remains incompletely defined, particularly in real-world clinical settings. Therefore, the aim of the present study was to evaluate the prognostic value of inflammatory status, assessed through SII, CRP, and NLR, together with NT-proBNP levels, in predicting adverse cardiovascular outcomes in patients with acute coronary syndromes in a single-center cohort.

## 2. Materials and Methods

### 2.1. Study Design

This prospective observational study included patients presenting with suspected acute coronary syndromes who met predefined criteria for systemic inflammation and were consecutively admitted to the Cardiology Department, Acute Coronary Care Unit of the Institute of Cardiovascular Diseases, Timișoara, Romania, between February 2024 and September 2025. During the study period, a total of 116 patients were screened for eligibility. After applying the inclusion and exclusion criteria, 100 patients with ACS were included in the final analysis. Reasons for non-inclusion included active malignancies (n = 3), known autoimmune or systemic inflammatory diseases (n = 2), and acute infections or myopericarditis (n = 6). Additional exclusions were due to incomplete clinical or laboratory data or lack of informed consent. Inflammatory status was defined as elevated levels of the inflammatory biomarkers assessed in this study, including C-reactive protein (CRP), neutrophil-to-lymphocyte ratio (NLR), and systemic immune-inflammation index (SII). Acute heart failure was defined as either present at admission or newly developed during hospitalization, requiring escalation of therapy, and was included as part of the in-hospital MACE endpoint.

**Figure 1:**
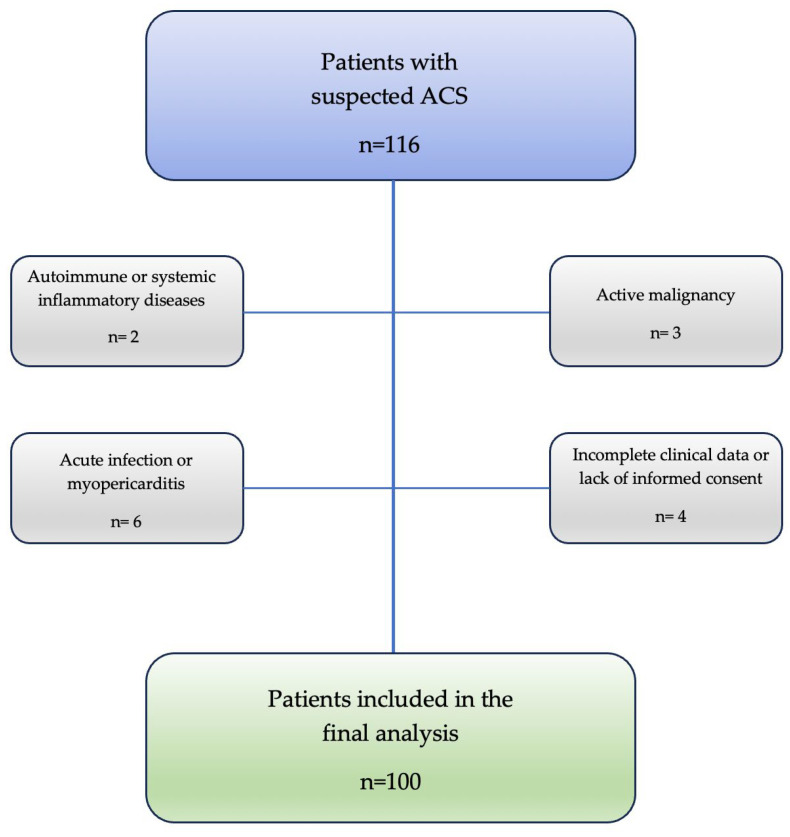
Study flow diagram. Out of 116 patients with suspected ACS screened for eligibility, 100 patients were included in the final analysis. Exclusion criteria included autoimmune or systemic inflammatory diseases (n = 2), active malignancy (n = 3), acute infection or myopericarditis (n = 6), and incomplete clinical data or lack of informed consent (n = 4).

The diagnosis of acute coronary syndromes (ACS), including unstable angina, NSTEMI, and STEMI, was established according to current European Society of Cardiology (ESC) guidelines and the Fourth Universal Definition of Myocardial Infarction [1,6]. Myocardial infarction cases were further classified as STEMI or NSTEMI based on electrocardiographic findings [7,8]. The median length of index hospitalization was 6 [4,5,6,7,8,9] days (Figure 1).

### 2.2. Data Collection and Clinical Assessment

Clinical and demographic data were obtained from the patients’ medical records and the institutional electronic database. The collected variables included demographic characteristics such as age, sex, and smoking status, as well as cardiovascular risk factors including arterial hypertension, diabetes mellitus, and dyslipidemia. Clinical parameters recorded at admission included heart rate, systolic and diastolic blood pressure, and body mass index (BMI). Cardiovascular risk factors were defined according to current European clinical guidelines [9,10]. Arterial hypertension was diagnosed based on the 2024 ESC/ESH Guidelines for the management of arterial hypertension, defined as an office blood pressure ≥ 140/90 mmHg or ongoing antihypertensive treatment [11]. Dyslipidemia was defined according to the 2019 ESC/EAS Guidelines for the management of dyslipidemias, as failure to achieve recommended lipid targets according to the patient’s cardiovascular risk category or the use of lipid-lowering therapy [12]. Diabetes mellitus was defined according to the 2023 ESC Guidelines for cardiovascular disease in patients with diabetes, based on a documented history of diabetes, the use of glucose-lowering medication, or laboratory criteria consistent with diabetes [13]. Smoking status was categorized as current smoker or non-smoker. Current smokers were defined as individuals who actively smoked at the time of hospital admission, while non-smokers included patients who had never smoked or had stopped smoking prior to admission [14].

### 2.3. Laboratory and Biomarker Assessment

Blood samples were collected from all patients at the time of hospital admission. Most laboratory parameters were obtained as part of the routine clinical evaluation; however, certain biomarkers were measured specifically for the purposes of the present study.

The assessed laboratory parameters included C-reactive protein, N-terminal pro–B-type natriuretic peptide, leukocyte count, neutrophil count, lymphocyte count, monocyte count, platelet count, fasting plasma glucose and serum creatinine levels. Renal function was evaluated by using serum creatinine and the estimated glomerular filtration rate (eGFR), expressed in mL/min/1.73 m^2^. The eGFR was calculated using the online calculator available at medcalc.org (accessed on 10 October 2025).

Inflammatory status was further assessed using derived hematological indices. The neutrophil-to-lymphocyte ratio was calculated as the ratio between the absolute neutrophil and lymphocyte counts [15]. The systemic immune-inflammation index was calculated using the formula platelet count × neutrophil count/lymphocyte count [16]. These indices were used as markers of systemic inflammatory response in patients with acute coronary syndromes. All laboratory measurements were performed according to standard hospital laboratory protocols.

### 2.4. Electrocardiographic and Echocardiographic Assessment

A standard 12-lead electrocardiogram (ECG) was performed at the time of admission as part of the routine diagnostic assessment for acute coronary syndromes.

Transthoracic echocardiography was subsequently performed according to standard echocardiographic protocols, by using a Vivid IQ ultrasound system (GE Healthcare, Milwaukee, WI, USA) equipped with a 2.5 MHz phased-array transducer. The examinations were conducted according to standard echocardiographic protocols. Left ventricular systolic function was evaluated by measuring the left ventricular ejection fraction (LVEF), calculated using the modified Simpson’s biplane method [17,18].

### 2.5. Coronary Angiography and Revascularization

Coronary angiography was performed in all patients according to standard institutional protocols, with operators blinded to the study protocol. The procedure was carried out via radial or femoral vascular access, according to patient characteristics and operator experience. Angiographic images were obtained in multiple projections to allow adequate visualization of the coronary anatomy and identification of the culprit lesion. Decisions regarding percutaneous coronary intervention (PCI) strategy were made by the treating interventional cardiologist based on angiographic findings and the overall clinical status of the patient, in accordance with current guideline recommendations.

### 2.6. Study Endpoints

The primary endpoint of the study was the occurrence of major adverse cardiovascular events (MACE) during hospitalization. MACE was defined as a composite outcome including cardiovascular death, recurrent myocardial infarction, stroke, urgent coronary revascularization, or acute heart failure requiring escalation of therapy. Acute heart failure was defined as either present at admission or newly developed during hospitalization, requiring escalation of therapy, and was included as part of the in-hospital MACE endpoint.

Secondary outcomes included in-hospital mortality and the association between inflammatory markers and the severity of ACS presentation. Their prognostic value for clinical outcomes was further evaluated using regression and discrimination analyses.

### 2.7. Ethical Considerations

The study was conducted in accordance with the principles of the Declaration of Helsinki and was approved by the Institutional Ethics Committee of “Victor Babeș” University of Medicine and Pharmacy Timișoara (approval code: 54, date: 2 October 2023) and the Institute of Cardiovascular Diseases Timișoara (approval code: 438, date: 22 January 2024). Written informed consent was obtained from all participants prior to inclusion in the study.

### 2.8. Statistical Analysis

Statistical analyses were conducted to assess the association between inflammatory markers and clinical outcomes in patients with acute coronary syndromes. Demographic and clinical variables, including age, sex, body mass index (BMI), cardiovascular risk factors (hypertension, diabetes mellitus, and smoking status), and clinical parameters at admission, were included in the descriptive and comparative analyses. In-hospital complications, including new-onset atrial fibrillation (NOAF), were also recorded and analyzed descriptively. Continuous variables were expressed as median and interquartile range (IQR), while categorical variables were presented as absolute numbers and percentages. The distribution of continuous variables was assessed using visual inspection and the Shapiro–Wilk test. Because most variables showed non-normal distributions, non-parametric statistical methods were applied. Comparisons between multiple groups were performed using the Kruskal–Wallis test for continuous variables and the chi-square test or Fisher’s exact test for categorical variables, as appropriate. For comparisons between two groups (e.g., patients with and without MACE), the Mann–Whitney U test was used for continuous variables. The inflammatory markers evaluated in the analysis included C-reactive protein, neutrophil-to-lymphocyte ratio, and the systemic immune-inflammation index. Because of skewed distributions, these variables were analyzed both in their original form and after logarithmic transformation when appropriate. Univariable logistic regression analyses were performed to estimate odds ratios (ORs) with 95% confidence intervals (CIs) for associations between potential predictors and MACE. Candidate predictors included age, sex, ACS subtype, renal function assessed by estimated glomerular filtration rate, left ventricular ejection fraction, and inflammatory markers. Multivariable logistic regression models were subsequently constructed to identify independent predictors of MACE and to assess the incremental prognostic value of inflammatory markers beyond baseline clinical variables. Variables included in the multivariable models were selected based on clinical relevance and with the aim of minimizing collinearity, particularly among variables reflecting overlapping pathophysiological processes. Additional sensitivity analyses were performed using tertile-based categorization of inflammatory markers to explore potential non-linear relationships between inflammatory burden and clinical outcomes. Model discrimination was evaluated using receiver operating characteristic (ROC) curve analysis, and predictive performance was summarized using the area under the curve (AUC). All tests were two-sided, and statistical significance was defined as *p* < 0.05. Data analysis was performed using R version 4.5.0 within the RStudio environment (version 2025.5.0.496) within the RStudio environment.

## 3. Results

A total of 116 patients with suspected acute coronary syndromes were screened for eligibility during the study period. After applying the inclusion and exclusion criteria, 100 patients were included in the final analysis. Among these, 81 patients presented with ST-segment elevation myocardial infarction, 12 with non–ST-segment elevation myocardial infarction, and 7 with unstable angina. Regarding the components of the composite MACE endpoint, stroke occurred in 3 patients (6%), urgent coronary revascularization in 4 (8%), recurrent myocardial infarction in 2 (4%), cardiovascular death in 6 (12%), and acute heart failure requiring escalation of therapy in 33 patients (66%).

### 3.1. Clinical Profile, Inflammatory Markers, and Admission Severity According to ACS Subtype

**Figure 2:**
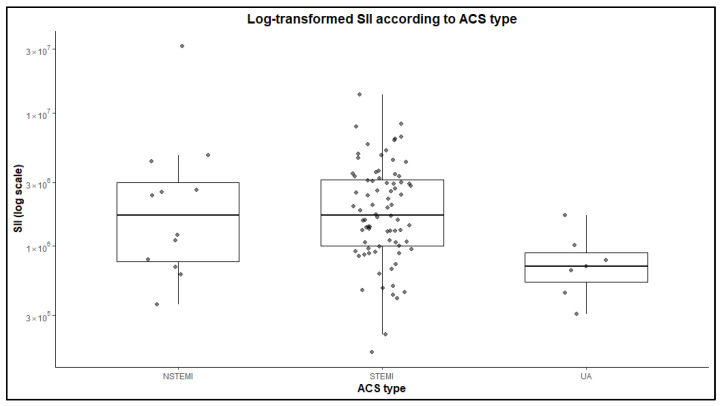
Distribution of log-transformed systemic immune-inflammation index (SII) according to ACS subtype. Higher SII values were observed in STEMI and NSTEMI compared with unstable angina.

The comparison of clinical and laboratory parameters across ACS subtypes revealed largely similar baseline characteristics among groups (Table 1 and Figure 2). Age, body mass index, systolic blood pressure, heart rate, renal function parameters, NT-proBNP levels, and left ventricular ejection fraction did not differ significantly between NSTEMI, STEMI, and unstable angina patients (all *p* > 0.05), indicating a similar clinical profile at the time of admission. Regarding inflammatory markers, leukocyte counts tended to be higher in myocardial infarction groups compared with unstable angina, although this difference did not reach statistical significance. In contrast, neutrophil counts differed significantly across ACS subtypes (*p* = 0.016), with the highest values observed in STEMI and NSTEMI patients. Similarly, the SII was significantly elevated in myocardial infarction compared with unstable angina (*p* = 0.017). Median SII values were markedly higher in both STEMI and NSTEMI, indicating a greater inflammatory burden in infarction presentations. The neutrophil-to-lymphocyte ratio also showed higher median values in myocardial infarction compared with unstable angina, although this association approached, but did not reach statistical significance (*p* = 0.057).

The distribution of categorical clinical characteristics across ACS subtypes showed generally similar demographic profiles and comorbidity burdens among groups (Table 2). Sex distribution, prevalence of hypertension, occurrence of new-onset atrial fibrillation, all-cause in-hospital mortality, and MACE did not differ significantly between NSTEMI, STEMI, and unstable angina patients (all *p* > 0.05), suggesting comparable baseline cardiovascular risk and management strategies across the cohort.

In contrast, diabetes mellitus showed a significant association with ACS subtype (*p* = 0.036), being more frequent among NSTEMI and unstable angina patients and less common in STEMI presentations. Similarly, smoking status differed significantly between groups (*p* = 0.032), with active smoking most prevalent among STEMI patients and less frequent in unstable angina. Although mortality appeared numerically higher among NSTEMI patients, the difference did not reach statistical significance. Likewise, the incidence of MACE was similar across ACS subtypes, indicating that short-term adverse outcomes were not strongly associated with clinical presentation alone.

### 3.2. Inflammatory Markers and Their Association with In-Hospital Outcomes in ACS/Inflammatory Markers and In-Hospital Outcomes in ACS

**Figure 3:**
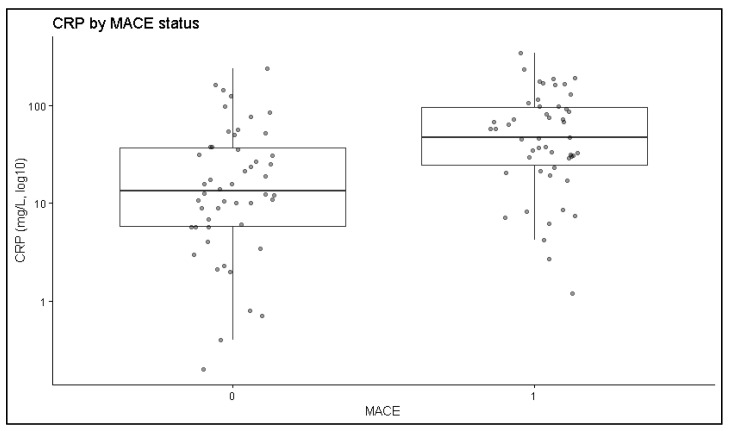
Distribution of Log-transformed C-reactive protein levels according to MACE occurrence. Higher logCRP values were observed among patients with MACE compared with those without events.

Significant differences were observed between patients who developed major adverse cardiovascular events and those without events across several clinical, functional, and inflammatory parameters (Table 3 and Figure 3). Patients who experienced MACE were significantly older (*p* = 0.003) and presented higher CRP values (*p* < 0.001) and NT-proBNP levels (*p* = 0.002) at admission, as illustrated in Figure 3. Composite inflammatory indices also differed between groups. The neutrophil-to-lymphocyte ratio (NLR, *p* = 0.030) and the systemic immune-inflammation index (SII, *p* = 0.042) were significantly higher in patients with MACE. Neutrophil counts tended to be higher, and lymphocyte counts lower, among these patients, although these differences did not reach statistical significance. Patients with MACE also had significantly lower left ventricular ejection fraction (*p* = 0.001) and reduced renal function, reflected by lower eGFR values (*p* < 0.001). Regarding comorbidities, hypertension was significantly more prevalent in the MACE group (*p* = 0.028). Diabetes appeared more frequently in patients with MACE, but did not reach statistical significance. Sex distribution and smoking status rates were comparable between groups. Separately, new-onset atrial fibrillation, recorded as an in-hospital complication, occurred significantly more frequently in patients with MACE (68% vs. 0%, *p* < 0.001).

The occurrence of major adverse cardiovascular events did not differ across ACS subtypes. The distribution of NSTEMI, STEMI, and unstable angina was similar between patients with and without MACE (*p* = 1.000), indicating that ACS subtype alone did not discriminate short-term outcomes in this cohort. In-hospital mortality occurred in a small proportion of patients, and no clinical or laboratory variable showed a statistically significant association with fatal outcomes. Age, inflammatory biomarkers, cardiac function, renal function, and comorbidities were comparable between survivors and non-survivors (all *p* > 0.05). Nevertheless, non-survivors showed numerically higher CRP levels, leukocyte and neutrophil counts, and NT-proBNP concentrations, along with lower lymphocyte counts, reduced left ventricular ejection fraction, and lower eGFR values. Higher median NLR and SII values were also observed among patients who experienced fatal in-hospital outcomes, although these differences did not reach statistical significance. The association between NLR and mortality approached statistical significance (*p* = 0.075). Regarding comorbidities, hypertension was present in all patients who experienced in-hospital mortality, and atrial fibrillation appeared more frequent in this group, although the differences were not statistically significant (Table 4).

The distribution of ACS subtypes did not differ significantly between patients with and without in-hospital mortality (*p* = 0.202). Although NSTEMI appeared proportionally more frequent among patients with in-hospital mortality, and unstable angina was not observed in this group, these differences did not reach statistical significance (Table 5).

### 3.3. Multivariable Analysis of Clinical and Inflammatory Predictors of MACE

**Figure 4:**
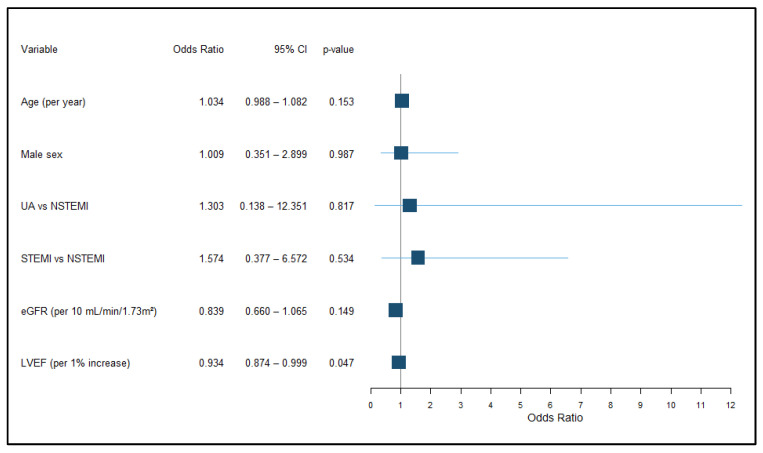
Forest plot of multivariable logistic regression analysis for MACE occurrence. Abbreviations: CI—confidence interval; UA—unstable angina; NSTEMI—non–ST-segment elevation myocardial infarction; STEMI—ST-segment elevation myocardial infarction; eGFR—estimated glomerular filtration rate; LVEF—left ventricular ejection fraction. ORs are expressed per unit increase for continuous variables.

In the multivariable logistic regression model, performed to identify independent predictors of major adverse cardiovascular events, and adjusted for demographic, clinical, and functional parameters, left ventricular ejection fraction emerged as the only independent predictor of major adverse cardiovascular events, underlying that each 1% increase in LVEF was associated with lower odds of adverse events (OR 0.934, 95% CI 0.874–0.999, *p* = 0.047) (Figure 4). Other variables included in the model were not independently associated with MACE. Age and renal function showed trends toward association with outcomes, with older age and lower eGFR linked to higher event rates; however, these relationships did not remain significant after adjustment. ACS subtype and sex were also not independently associated with MACE.

When C-reactive protein was added to the baseline multivariable model (Table 6), it showed a modest association with MACE occurrence, although this did not reach statistical significance (OR 1.008 per mg/L, 95% CI 0.999–1.018, *p* = 0.098). In this expanded model, none of the variables retained independent statistical significance. The association previously observed for left ventricular ejection fraction was attenuated after inclusion of CRP.

When the neutrophil-to-lymphocyte ratio (NLR) was incorporated into the multivariable model (Table 7), it was not independently associated with MACE (OR 1.014 per unit, 95% CI 0.959–1.072, *p* = 0.618). As in the previous model, none of the covariates reached statistical significance, although left ventricular ejection fraction showed a borderline association with outcomes (*p* = 0.054).

Similarly, SII was not independently associated with MACE when included in the multivariable model (Table 8). Left ventricular ejection fraction remained the only variable independently associated with outcomes after adjustment.

Interaction analyses showed no significant interaction between inflammatory markers and STEMI presentation (Table 9). In models including CRP, NLR, and SII, neither the markers nor their interaction terms were significantly associated with MACE after adjustment.

### 3.4. Evaluation of Predictive Performance and Incremental Value of Inflammatory Markers

The baseline multivariable model demonstrated good discriminative ability for predicting MACE, with an AUC of 0.775 (95% CI 0.682–0.868). The addition of inflammatory markers resulted in only modest changes in predictive performance.

**Figure 5:**
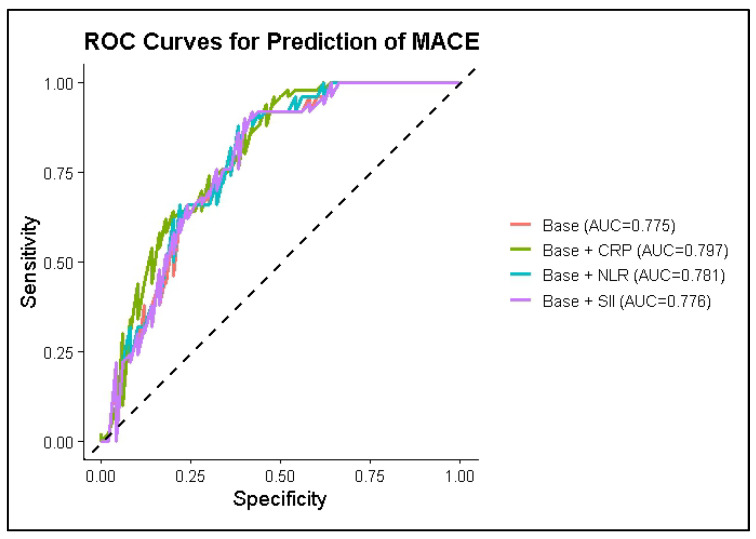
ROC curves illustrating the discriminative performance of the baseline multivariable model and models additionally including CRP, NLR and SII for the prediction of major adverse cardiovascular events.

Receiver operating characteristic (ROC) curves analysis showed that inclusion of CRP produced the largest numerical improvement in model performance, increasing the AUC to 0.797, whereas models including NLR and SII yielded AUC values of 0.781 and 0.776, respectively. The corresponding ROC curves are shown in Figure 5, and model discrimination metrics are reported in Table 10.

However, formal comparison of ROC curves using the DeLong test indicated that the addition of inflammatory markers was not associated with a statistically significant change in discriminative performance (all *p* > 0.05).

### 3.5. Sensitivity Analyses of Inflammatory Markers and Their Association with MACE

Sensitivity analyses using tertile-based categorization and log-transformed models are presented in Table 11. Higher CRP levels were associated with an increased risk of MACE. Patients in the highest CRP tertile had significantly higher odds of events compared with those in the lowest tertile (OR 4.47, *p* = 0.023), and a significant trend across tertiles was observed (*p* = 0.023). In addition, log-transformed CRP showed a significant association with MACE (OR 1.67, *p* = 0.017). For NLR, the intermediate tertile showed a borderline association with MACE (*p* = 0.073), whereas the highest tertile, trend analysis, and log-transformed models were not statistically significant. For SII, patients in the second tertile had significantly higher odds of MACE (OR 5.48, *p* = 0.010); however, this association was not observed in the highest tertile, and neither the trend analysis nor the log-transformed model reached statistical significance.

## 4. Discussion

The present study evaluated the prognostic significance of inflammatory status in patients presenting with acute coronary syndromes in a single-center cohort. The findings suggest that systemic inflammation is associated with short-term adverse outcomes in this population, with higher levels of inflammatory markers observed among patients who developed major adverse cardiovascular events during hospitalization. Among the biomarkers evaluated, C-reactive protein demonstrated the most robust association with adverse outcomes in exploratory and sensitivity analyses, while the composite inflammatory indices NLR and SII also showed associations with MACE. After adjustment for clinical and functional parameters, the strength of these associations was attenuated, suggesting that inflammatory biomarkers may reflect the systemic inflammatory response accompanying acute coronary syndromes. Importantly, left ventricular ejection fraction emerged as an independent predictor of in-hospital MACE, highlighting the central role of ventricular systolic dysfunction in determining short-term prognosis in patients with ACS. The addition of inflammatory markers to the baseline clinical model resulted in only modest and statistically nonsignificant improvements in predictive performance.

Systemic inflammation plays a fundamental role in the pathophysiology of atherosclerosis and acute coronary syndromes. Inflammatory activation contributes to endothelial dysfunction, progression of atherosclerotic plaques, and ultimately plaque destabilization and thrombus formation, leading to myocardial ischemia [19]. In this context, circulating inflammatory biomarkers have attracted increasing attention as potential tools for risk stratification in patients with ACS. Among these, C-reactive protein has been extensively studied and consistently associated with plaque instability, increased thrombotic activity, and adverse cardiovascular outcomes [20,21]. Our findings support this concept, as higher CRP levels were observed among patients who developed major adverse cardiovascular events during hospitalization, and sensitivity analyses demonstrated a graded relationship between increasing CRP levels and the risk of MACE. In addition to CRP, composite inflammatory indices derived from routine hematological parameters have recently gained attention as potential prognostic markers in cardiovascular disease. The neutrophil-to-lymphocyte ratio and the systemic immune-inflammation index integrate information on different components of the immune response and have been proposed as indicators of systemic inflammatory activation and immune imbalance [22]. Previous studies have reported associations between elevated NLR or SII values and adverse cardiovascular outcomes in patients with acute coronary syndromes [23,24]. In the present study, both NLR and SII were higher among patients who experienced MACE, supporting the concept that systemic inflammatory activation accompanies more severe clinical presentations of acute coronary syndromes. These composite indices integrate information from different components of the immune response and may therefore capture the complex interplay between inflammation, immune imbalance, and thrombotic activity during the acute phase of coronary events. After adjustment for clinical and functional variables, the strength of these associations appeared attenuated, suggesting that NLR and SII may primarily reflect the broader inflammatory burden accompanying ACS rather than acting as independent determinants of short-term outcomes.

Left ventricular systolic function is a well-established determinant of prognosis in patients with acute coronary syndromes. Reduced left ventricular ejection fraction reflects the extent of myocardial injury and the degree of hemodynamic compromise, both of which are strongly associated with adverse clinical outcomes [25,26]. In our research, LVEF emerged as the only independent predictor of in-hospital MACE after adjustment for demographic, clinical, and inflammatory variables. Each 1% increase in LVEF was associated with a lower probability of adverse events, highlighting the protective role of preserved ventricular systolic function, an observation reflecting the notable contribution of acute heart failure events to MACE in our cohort. This finding is consistent with previous studies demonstrating that impaired ventricular function remains one of the strongest predictors of short-term complications and mortality following acute coronary events. The persistence of LVEF as an independent determinant of outcome in our model suggests that ventricular systolic function reflects the overall severity of the acute ischemic event [27,28]. At the same time, inflammatory biomarkers may capture complementary aspects of the underlying pathophysiological processes, including systemic inflammatory activation associated with acute coronary syndromes.

The findings of the present study should be interpreted with caution in terms of clinical applicability. Inflammatory biomarkers, including CRP, NLR, and SII, were associated with adverse outcomes and may reflect the underlying inflammatory status accompanying acute coronary events. Although their independent prognostic contribution was attenuated after adjustment for established clinical predictors, these markers may still provide complementary information regarding the systemic inflammatory response in ACS. When interpreted in the appropriate clinical context, inflammatory biomarkers may contribute to a more comprehensive characterization of disease severity. In this context, contemporary evidence emphasizes that prognosis in ACS is largely driven by clinical factors such as ventricular dysfunction, hemodynamic status, and comorbidity burden, while the incremental role of novel biomarkers remains an area of ongoing investigation. In this context, inflammatory biomarkers may provide additional pathophysiological insight but should be interpreted within the broader clinical framework [29].

### 4.1. Future Perspectives

The relationship between systemic inflammation and arrhythmic complications in patients with acute coronary syndromes represents an important area for further investigation. In the present cohort, new-onset atrial fibrillation emerged as a frequent in-hospital complication and was more commonly observed in patients who developed adverse cardiovascular events. Although the determinants of atrial fibrillation were not specifically explored in the current analysis, this observation highlights the potential relevance of arrhythmic complications in the clinical course of ACS. Growing evidence suggests that inflammatory activation may contribute to atrial electrical and structural remodeling, potentially favoring the occurrence of atrial fibrillation in the acute phase of coronary syndromes [30,31]. Further investigation of the relationship between inflammatory biomarkers and the development of new-onset atrial fibrillation in patients with ACS may provide additional insight into the mechanisms linking inflammation, myocardial injury, and arrhythmic complications, and represents an important direction for future research.

### 4.2. Limitations

The present study has several limitations that should be acknowledged. Although the study was prospectively conducted, it was performed in a single center with a relatively limited sample size, which may affect the generalizability of the findings. Second, the analysis focused on in-hospital outcomes, and therefore, the long-term prognostic implications of inflammatory biomarkers could not be evaluated. Third, inflammatory markers were primarily assessed at admission, and serial measurements were not systematically available throughout hospitalization. In addition, the relatively small number of patients with NSTEMI and unstable angina limits the statistical power of subgroup comparisons across ACS subtypes; therefore, interaction analyses involving STEMI should be considered exploratory and interpreted in the context of these limitations. Similarly, the small number of in-hospital deaths in our cohort limits the statistical power of mortality-related analyses and precludes robust inferential conclusions. As such, mortality findings should be interpreted as descriptive observations rather than definitive associations.

Finally, although several clinical and laboratory variables were included in the multivariable models, the possibility of residual confounding cannot be completely excluded. Larger multicenter studies are needed to further clarify the prognostic role of inflammatory biomarkers in patients with acute coronary syndromes.

## 5. Conclusions

In patients with acute coronary syndromes, the occurrence of major adverse cardiovascular events during index hospitalization, with a median length of stay of 6 days, was associated with an unfavorable clinical profile, characterized by older age, impaired renal function, and reduced left ventricular systolic function. Levels of inflammatory biomarkers (CRP, NLR, and SII) and NT-proBNP were significantly higher among those who developed in-hospital MACE compared with those without events, with CRP showing the strongest association. New-onset atrial fibrillation was also more frequently observed in the MACE group. In multivariable analysis, left ventricular ejection fraction emerged as an independent predictor of short-term outcomes.

Overall, these findings suggest that inflammatory biomarkers reflect the inflammatory status accompanying acute coronary syndromes and may provide complementary information regarding the inflammatory burden, while their incremental prognostic contribution beyond established clinical variables remains to be further clarified.

## Figures and Tables

**Table 1:** Baseline clinical and laboratory characteristics according to ACS subtype.

Variable	NSTEMI (n = 12)	STEMI (n = 81)	UA (n = 7)	*p*-Value
**Age, years**	72.00 [65.75–77.25]	63.00 [54.00–73.00]	69.00 [64.00–74.00]	0.209
**BMI, kg/m^2^**	27.80 [26.35–29.70]	28.00 [25.35–31.20]	26.00 [25.20–29.24]	0.783
**SBP, mmHg**	135.00 [100.00–160.00]	130.00 [115.00–145.00]	135.00 [122.50–147.50]	0.914
**Heart rate, bpm**	89.50 [71.50–100.00]	80.00 [70.00–98.00]	77.00 [73.50–79.00]	0.634
**CRP, mg/L**	18.00 [14.45–64.55]	31.70 [10.10–77.00]	9.00 [3.50–29.70]	0.241
**NT-proBNP, pg/mL**	1642.95 [1020.62–5882.52]	2350.40 [1090.00–6840.00]	1386.20 [836.70–4154.55]	0.394
**Leukocytes, µL**	13,290.00 [9652.50–15,585.00]	12,860.00 [10,585.00–16,000.00]	9240.00 [7350.00–12,870.00]	0.165
**Neutrophils, µL**	9930.00 [7117.50–13,210.00]	10,657.00 [7810.00–12,847.00]	6780.00 [4240.00–7775.00]	**0.016**
**Lymphocytes, µL**	1555.00 [1197.50–2517.50]	1500.00 [1100.00–2030.00]	1880.00 [1285.00–2325.00]	0.773
**Monocytes, µL**	665.00 [595.00–905.00]	690.00 [520.00–920.00]	500.00 [490.00–605.00]	0.340
**Platelets, µL**	291,000.00 [213,750.00–324,750.00]	260,000.00 [218,000.00–309,000.00]	215,000.00 [188,500.00–224,000.00]	0.065
**NLR**	6.33 [3.36–10.02]	7.04 [4.39–10.87]	3.41 [2.47–4.61]	0.057
**SII**	1,811,454.74 [763,419.58–3,066,110.07]	1,729,378.64 [999,480.72–3,101,054.54]	699,058.58 [548,578.30–895,010.28]	**0.017**
**eGFR, mL/min/1.73 m^2^**	50.40 [46.10–55.45]	66.00 [44.03–81.12]	69.27 [62.39–70.60]	0.544
**Creatinine, mg/dL**	1.15 [1.07–1.60]	1.15 [0.97–1.58]	1.02 [0.86–1.23]	0.283
**LVEF, %**	40.00 [30.00–40.00]	39.00 [32.00–45.00]	45.00 [32.50–45.00]	0.712

Abbreviations: BMI—body mass index; SBP—systolic blood pressure; CRP—C-reactive protein; NT-proBNP—N-terminal pro–B-type natriuretic peptide; NLR—neutrophil-to-lymphocyte ratio; SII—systemic immune-inflammation index; eGFR—estimated glomerular filtration rate; LVEF—left ventricular ejection fraction.

**Table 2:** Categorical clinical characteristics according to ACS subtype.

Variable	NSTEMI (n = 12)	STEMI (n = 81)	UA (n = 7)	*p*-Value
**Male sex**	7 (58.3%)	56 (69.1%)	6 (85.7%)	0.499
**Hypertension**	8 (66.7%)	57 (70.4%)	6 (85.7%)	0.769
**Diabetes**	7 (58.3%)	22 (27.2%)	4 (57.1%)	**0.036**
**Smoker**	5 (41.7%)	50 (61.7%)	1 (14.3%)	**0.032**
**NOAF**	5 (41.7%)	26 (32.1%)	3 (42.9%)	0.662
**In-hospital mortality**	2 (16.7%)	4 (4.9%)	0 (0.0%)	0.202
**MACE**	6 (50.0%)	41 (50.6%)	3 (42.9%)	1.000

Abbreviations: STEMI—ST-segment elevation myocardial infarction; NSTEMI—non–ST-segment elevation myocardial infarction; UA—unstable angina; NOAF—new onset atrial fibrillation; MACE—major adverse cardiovascular events.

**Table 3:** Baseline predictors of MACE occurrence in patients with acute coronary syndromes.

Variable	No MACE (n = 50)	MACE (n = 50)	*p*-Value
**Age (years)**	58.50 [50.25–71.75]	69.00 [61.25–77.00]	**0.003**
**CRP (mg/L)**	13.30 [5.78–36.90]	46.90 [24.53–96.75]	**<0.001**
**NT-proBNP (pg/mL)**	1456.45 [953.60–3215.88]	3522.70 [1904.38–8007.00]	**0.002**
**Leukocytes (/µL)**	11,716.00 [9145.00–16,300.00]	13,460.00 [11,500.00–15,647.50]	0.152
**Neutrophils (/µL)**	9105.00 [6562.50–13,738.50]	11,460.00 [8595.00–12,712.50]	0.081
**Lymphocytes (/µL)**	1770.00 [1200.00–2185.00]	1330.00 [962.50–1930.00]	0.065
**NLR**	4.78 [3.12–9.64]	7.47 [4.67–10.22]	**0.030**
**SII**	1,206,896.15 [715,241.35–2,941,096.11]	1,897,722.94 [1,280,874.26–3,171,769.47]	**0.042**
**LVEF (%)**	40.00 [35.00–45.00]	35.00 [30.00–40.00]	**0.001**
**eGFR (mL/min/1.73 m^2^)**	72.26 [50.84–90.66]	47.85 [30.28–68.75]	**<0.001**
**Male sex**	36 (72.0%)	33 (66.0%)	0.665
**Hypertension**	30 (60.0%)	41 (82.0%)	**0.028**
**Diabetes**	12 (24.0%)	21 (42.0%)	0.089
**Current smoker**	30 (60.0%)	26 (52.0%)	0.546

Abbreviations: MACE—major adverse cardiovascular events; CRP—C-reactive protein; NT-proBNP—N-terminal pro–B-type natriuretic peptide; NLR—neutrophil-to-lymphocyte ratio; SII—systemic immune-inflammation index; LVEF—left ventricular ejection fraction; eGFR—estimated glomerular filtration rate; NOAF—new onset atrial fibrillation.

**Table 4:** Baseline predictors of in-hospital death in patients with acute coronary syndromes.

Variable	Survivors (n = 94)	In-Hospital Mortality (n = 6)	*p*-Value
**Age (years)**	64.50 [54.00–75.00]	63.50 [62.00–74.75]	0.586
**CRP (mg/L)**	29.65 [9.25–71.38]	65.75 [28.57–71.20]	0.349
**NT-proBNP (pg/mL)**	2152.35 [1004.35–6774.73]	2741.30 [1985.37–6918.35]	0.357
**Leukocytes (/µL)**	12,795.00 [10,052.50–15,952.50]	14,270.00 [12,995.00–14,982.50]	0.464
**Neutrophils (/µL)**	9550.00 [7325.00–12,846.00]	11,740.00 [10,925.00–12,607.50]	0.220
**Lymphocytes (/µL)**	1535.00 [1100.00–2220.00]	1290.00 [1185.00–1470.00]	0.215
**NLR**	5.88 [3.54–10.17]	9.51 [8.58–10.73]	0.075
**SII**	1,492,725.67 [881,587.28–3,055,319.67]	2,549,059.66 [2,430,531.20–2,633,606.78]	0.106
**LVEF (%)**	40.00 [32.75–45.00]	32.50 [30.00–35.00]	0.118
**eGFR (mL/min/1.73 m^2^)**	62.96 [39.01–82.61]	48.36 [42.08–63.84]	0.286
**Male sex**	65 (69.1%)	4 (66.7%)	1.000
**Hypertension**	65 (69.1%)	6 (100.0%)	0.177
**Diabetes**	30 (31.9%)	3 (50.0%)	0.393
**Current smoker**	53 (56.4%)	3 (50.0%)	1.000
**NOAF**	30 (31.9%)	4 (66.7%)	0.176

Abbreviations: CRP—C-reactive protein; NT-proBNP—N-terminal pro–B-type natriuretic peptide; NLR—neutrophil-to-lymphocyte ratio; SII—systemic immune-inflammation index; LVEF—left ventricular ejection fraction; eGFR—estimated glomerular filtration rate; NOAF—new-onset atrial fibrillation.

**Table 5:** Association between ACS subtype and in-hospital mortality.

Variable	Level	Survivors (n = 94)	Death (n = 6)	*p*-Value
**ACS subtype**	NSTEMI	10 (10.6%)	2 (33.3%)	0.202
	STEMI	77 (81.9%)	4 (66.7%)	
	UA	7 (7.4%)	0 (0.0%)	

Abbreviations: ACS—acute coronary syndrome; NSTEMI—non–ST-segment elevation myocardial infarction; STEMI—ST-segment elevation myocardial infarction; UA—unstable angina.

**Table 6:** Multivariable logistic regression model for MACE including C-reactive protein.

Variable	OR	95% CI	*p*-Value
**Age (per year)**	1.031	0.984–1.080	0.196
**Male sex**	0.824	0.275–2.473	0.730
**UA vs. NSTEMI**	1.456	0.151–14.050	0.745
**STEMI vs. NSTEMI**	1.424	0.316–6.412	0.645
**eGFR (per 10 mL/min/1.73 m^2^)**	0.875	0.684–1.119	0.288
**LVEF (per 1% increase)**	0.945	0.882–1.012	0.106
**CRP (per 1 mg/L)**	1.008	0.999–1.018	0.098

Abbreviations: OR—odds ratio; CI—confidence interval; UA—unstable angina; NSTEMI—non–ST-segment elevation myocardial infarction; STEMI—ST-segment elevation myocardial infarction; eGFR—estimated glomerular filtration rate; LVEF—left ventricular ejection fraction; CRP—C-reactive protein. ORs are expressed per unit increase for continuous variables.

**Table 7:** Multivariable logistic regression model for MACE including neutrophil-to-lymphocyte ratio.

Variable	OR	95% CI	*p*-Value
**Age (per year)**	1.034	0.987–1.082	0.159
**Male sex**	0.999	0.347–2.879	0.999
**UA vs. NSTEMI**	1.422	0.145–13.987	0.763
**STEMI vs. NSTEMI**	1.647	0.381–7.112	0.504
**eGFR (per 10 mL/min/1.73 m^2^)**	0.847	0.665–1.077	0.176
**LVEF (per 1% increase)**	0.936	0.875–1.001	0.054
**NLR (per unit)**	1.014	0.959–1.072	0.618

Abbreviations: OR—odds ratio; CI—confidence interval; UA—unstable angina; NSTEMI—non–ST-segment elevation myocardial infarction; STEMI—ST-segment elevation myocardial infarction; eGFR—estimated glomerular filtration rate; LVEF—left ventricular ejection fraction; NLR—neutrophil-to-lymphocyte ratio. ORs are expressed per unit increase for continuous variables.

**Table 8:** Multivariable logistic regression model for MACE including systemic immune-inflammation index.

Variable	OR	95% CI	*p*-Value
**Age (per year)**	1.034	0.988–1.083	0.151
**Male sex**	1.014	0.352–2.916	0.980
**UA vs. NSTEMI**	1.266	0.131–12.262	0.839
**STEMI vs. NSTEMI**	1.551	0.369–6.510	0.549
**eGFR (per 10 mL/min/1.73 m^2^)**	0.837	0.658–1.065	0.147
**LVEF (per 1% increase)**	0.934	0.873–0.999	**0.047**
**SII (per 100,000 units)**	0.999	0.986–1.012	0.860

**Table 9:** Multivariable interaction models evaluating whether the prognostic impact of inflammatory markers differs by ACS presentation.

Variable	OR	95% CI	*p*-Value
**Model: Base + CRP + Interaction**			
**Age (per year)**	1.03	0.99–1.08	0.164
**LVEF (per 1%)**	0.93	0.87–1.01	0.066
**CRP (per 1 mg/L)**	1.10	0.98–1.23	0.101
**CRP × STEMI**	0.92	0.82–1.03	0.125
**Model: Base + NLR + Interaction**			
**Age (per year)**	1.03	0.98–1.08	0.195
**LVEF (per 1%)**	0.94	0.88–1.01	0.074
**NLR (per unit)**	1.29	0.75–2.24	0.358
**NLR × STEMI**	0.76	0.44–1.31	0.320
**Model: Base + SII + Interaction**			
**Age (per year)**	1.03	0.98–1.08	0.195
**LVEF (per 1%)**	0.94	0.87–1.00	0.058
**SII (per 10^5^ units)**	1.10	0.94–1.29	0.253
**SII × STEMI**	0.89	0.76–1.05	0.173

**Table 10:** Discriminative performance of multivariable models for prediction of MACE.

Model	AUC (95% CI)
**Base model**	0.775 (0.682–0.868)
**Base + CRP**	0.797 (0.708–0.885)
**Base + NLR**	0.781 (0.689–0.873)
**Base + SII**	0.776 (0.683–0.869)

Abbreviations: AUC—area under the curve; CI—confidence interval; CRP—C-reactive protein; NLR—neutrophil-to-lymphocyte ratio; SII—systemic immune-inflammation index.

**Table 11:** Sensitivity analyses of CRP, NLR, and SII using tertile and log-transformed models for the prediction of MACE.

Marker	Model	OR (95% CI)	*p*-Value
**CRP**	CRP tertile T2 vs. T1	2.54 (0.80–8.03)	0.113
	CRP tertile T3 vs. T1	4.47 (1.23–16.23)	**0.023**
	Trend per tertile increase	2.11 (1.11–4.02)	**0.023**
	logCRP (continuous)	1.67 (1.09–2.55)	**0.017**
**NLR**	NLR tertile T2 vs. T1	2.83 (0.91–8.85)	0.073
	NLR tertile T3 vs. T1	1.88 (0.56–6.32)	0.307
	Trend per tertile increase	1.40 (0.77–2.55)	0.271
	logNLR (continuous)	1.30 (0.61–2.77)	0.489
**SII**	SII tertile T2 vs. T1	5.48 (1.49–20.09)	**0.010**
	SII tertile T3 vs. T1	1.74 (0.49–6.19)	0.390
	Trend per tertile increase	1.21 (0.66–2.22)	0.540
	logSII (continuous)	1.08 (0.60–1.94)	0.794

Abbreviations: OR—odds ratio; CI—confidence interval; CRP—C-reactive protein; NLR—neutrophil-to-lymphocyte ratio; SII—systemic immune-inflammation index; T1–T3—tertiles of marker distribution.

## Data Availability

Data are available on request due to restrictions (privacy and ethical reasons).
